# Runx1: a new driver in neurofibromagenesis

**DOI:** 10.18632/oncoscience.266

**Published:** 2015-11-17

**Authors:** Jianqiang Wu, Gang Huang, Nancy Ratner

**Affiliations:** Division of Experimental Hematology and Cancer Biology, Cancer and Blood Diseases Institute, Cincinnati Children's Hospital Research Foundation, Cincinnati Children's Hospital, University of Cincinnati, Cincinnati, OH, USA

**Keywords:** Nf1, Runx1, neurofibroma, Schwann cells

The Runt-related transcription factor-1 (RUNX1 or AML1) encodes a transcription factor that serves as a master developmental regulator. It is important for hematopoiesis, angiogenesis, maturation of megakaryocytes, and differentiation of T and B cells [1]. Runx1 is also important for neuronal development and glial cell differentiation [2]. Runx1 is integrated into a complex regulatory network which acts both at the transcriptional and post-transcriptional levels. Runx1 activity can be regulated by several posttranslational modifications, including phosphorylation, de-phosphorylation, SUMOylation, acetylation, methylation and ubiquitination. These modifications control various aspects of transcriptional factors' activities such as auto inhibition, dimerization and ubiquitin-mediated degradation [3].

Besides its developmental determination role, *RUNX1* is involved in malignant tumor formation. Reports have shown that *RUNX1* is frequently de-regulated and has paradoxical effects in human cancers, in which it can function either as a tumor suppressor or oncogene [3, 4]. *RUNX1* has been implicated as a tumor suppressor in several solid tumors including breast cancer, esophageal adenocarcinoma, colon cancer and possibly prostate cancer but acts as an oncogene in head/neck squamous cell carcinomas, endometrial cancer, and epithelial cancer [3, 4]. Because Runx1 is a sequence specific DNA-binding transcription factor, whether it functions as oncogene or tumor suppressor is dependent on its interaction with specific co-regulatory proteins.

**Figure 1:**
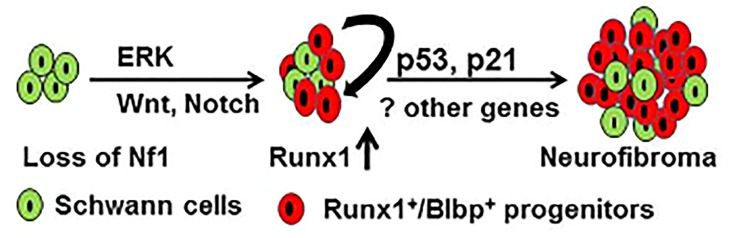
Model of neurofibromagenesis Loss of *Nf1* in Schwann cells (green) leads to activation of ERK, Wnt or Notch signaling pathways as well as increased number of Runx1^+^/Blbp^+^ Schwann cell progenitors (red). These progenitors proliferate, differentiate and self-renew, expanding the progenitor and Schwann cell populations by inhibiting p53/p21 or other unknown pathway(s) to form neurofibromas.

We recently showed that *RUNX1* acts as an oncogene in the context of loss of neurofibromatosis type 1 (*Nf1*). Instead of chromosomal translocation and mutation frequently detected in other cancers, Runx1 is overexpressed in human and mouse neurofibroma-initiating cells, both at the messenger RNA and protein levels. Specifically, loss of *Nf1* increases number of embryonic day 12.5 Runx1^+^/Blbp^+^ Schwann cell progenitors that enable neurofibroma formation in a mouse model (Figure 1). Targeted genetic deletion of *RUNX1* in Schwann cells and Schwann cell progenitors delays mouse neurofibroma formation *in vivo* (5).

It is not clear how loss of *Nf1* induces *Runx1* overexpression and serves as an oncogene. There are several potential possibilities: 1) *NF1* is known to encode a Ras-GTPase activating protein (Ras-GAP) and the Ras-MEK-ERK pathway is important for Nf1 neurofibroma formation [6]. Runx1 may be phosphorylated by the elevated MEK signaling to initiate the tumor formation process. 2) Elevated Wnt or Notch signaling can directly or indirectly activate Runx1, which can accelerate G1-S transition and stimulates cell proliferation. Consistently, our results show that loss of Runx1 in Schwann cells decreased cell proliferation by activating Trp53-p21 or increased cell apoptosis by inhibiting anti-apoptotic gene Bcl-2 in the context of *Nf1−/−* Schwann cell environment. It is possible that the elevated Runx1 within the neurofibroma cell alters cell fate (i.e. proliferation or differentiation) through Trp53-p21. Further experiments are needed to determine how p21 and Trp53 were activated or Bcl-2 was inactivated in the *Runx1^fl/fl^;Nf1^fl/fl^; Dhhre* tumors. 3) Runx1 might interact with epigenetic regulators such as the chromatin remodeling complexes, SWI/SNF, or polycomb repressive complexes to affect Runx1 activity by post-translational modification [7].

Overall, our study supports an oncogenic role of Runx1 in neurofibroma initiation and/or maintenance, but the underlying mechanism(s) are not clear. With increasing knowledge of RUNX1 biology, targeting the transcription factor RUNX1 or *RUNX1* pathway might provide a novel therapy for neurofibroma as well as other tumors which overexpress *RUNX1*.
